# Soybean iron deficiency chlorosis high throughput phenotyping using an unmanned aircraft system

**DOI:** 10.1186/s13007-019-0478-9

**Published:** 2019-08-20

**Authors:** Austin A. Dobbels, Aaron J. Lorenz

**Affiliations:** Department of Agronomy and Plant Genetics, University of Minnesota, 1991 Upper Buford Circle, 411 Borlaug Hall, St. Paul, MN 55108 USA

**Keywords:** Unmanned aircraft system (UAS), Soybean, Iron deficiency chlorosis, Remote sensing, Random forest, Neural network, Image analysis

## Abstract

**Background:**

Iron deficiency chlorosis (IDC) is an abiotic stress in soybean [Glycine max (L.) Merr.] that causes significant yield reductions. Symptoms of IDC include interveinal chlorosis and stunting of the plant. While there are management practices that can overcome these drastic yield losses, the preferred way to manage IDC is growing tolerant soybean varieties. To develop varieties tolerant to IDC, breeders may easily phenotype up to thousands of candidate soybean lines every year for severity of symptoms related to IDC, a task traditionally done with a 1–5 visual rating scale. The visual rating scale is subjective and, because it is time consuming and laborious, can typically only be accomplished once or twice during a growing season.

**Results:**

The goal of this study was to use an unmanned aircraft system (UAS) to improve field screening for tolerance to soybean IDC. During the summer of 2017, 3386 plots were visually scored for IDC stress on two different dates. In addition, images were captured with a DJI Inspire 1 platform equipped with a modified dual camera system which simultaneously captures digital red, green, blue images as well as red, green, near infrared (NIR) images. A pipeline was created for image capture, orthomosaic generation, processing, and analysis. Plant and soil classification was achieved using unsupervised classification resulting in 95% overall classification accuracy. Within the plant classified canopy, the green, yellow, and brown plant pixels were classified and used as features for random forest and neural network models. Overall, the random forest and neural network models achieved similar misclassification rates and classification accuracy, which ranged from 68 to 77% across rating dates. All 36 trials in the field were analyzed using a linear model for both visual score and UAS predicted values on both dates. In 32 of the 36 tests on date 1 and 33 of 36 trials on date 2, the LSD associated with UAS image-based IDC scores was lower than the LSD associated with visual scores, indicating the image-based scores provided more precise measurements of IDC severity.

**Conclusions:**

Overall, the UAS was able to capture differences in IDC stress and may be used for evaluations of candidate breeding lines in a soybean breeding program. This system was both more efficient and precise than traditional scoring methods.

## Background

Iron deficiency Chlorosis (IDC) is a major soil borne stress in soybean [Glycine max (L.) Merr.] and causes significant yield reductions. In the United States, soybean IDC has been reported to result in yield losses totaling $260 million annually [1, 2]. Soybean IDC is caused by a lack of available iron [Fe(II)] to the plant [3–6]. While iron is abundant in almost all soils, deficiencies are caused by several soil chemical factors and their interactions that change the solubility of iron in the soil [2, 7]. Soybean IDC is impacted by soil pH, soil calcium carbonate content, soil moisture content, soil electrical conductivity, iron oxide concentration, and soluble salts [2, 8–10]. Deficiency symptoms include interveinal chlorosis and overall stunting of the plant [11, 12]. Soybean growers can overcome the drastic yield penalties of IDC by growing tolerant soybean varieties, planting companion crops, reducing other forms of plant stress, and supplementing the soil with iron chelates [13, 14].

The preferred method to minimize yield losses caused by IDC is growing a tolerant variety, which is why there is continued interest in the development of IDC tolerant varieties by soybean breeders [13, 15, 16]. To accomplish this breeding objective, thousands of potential soybean varieties need to be screened every year for IDC severity. The screening has traditionally been accomplished using a 1–5 visual severity scoring system where a score of 1 is given to tolerant lines and a score of 5 is given to susceptible lines [6]. The visual rating system is labor intensive and typically only done at one time point in the growing season. In addition, intra-rater variability due to the subjectivity of the human eye can result in less accurate phenotypic measurements, and thus, researchers are investigating image-based methods for quantifying IDC severity [6, 17]. New automated rating systems hold potential for more objectivity and reliability for phenotyping IDC stress [17, 18]. To date, these phenotyping methods have been implemented from tripods [17] and push carts [19]; however, implementation using an unmanned aircraft system (UAS) has not been reported.

High throughput phenotyping (HTP) refers to the ability for researchers to collect detailed information during a plant life cycle in a non-invasive way [20, 21]. This can be done in both controlled and field environments and with a wide array of platforms and sensors including ground based and aerial systems [18, 20]. In recent years, there has been a growing interest in the utilization of aerial HTP platforms, especially for use in germplasm assessment within breeding programs [22]. While many traits are currently being measured in soybean using these platforms, including plant maturity, canopy coverage, and yield estimation [23, 24], for example, the main goal of this project was to use an unmanned aircraft system (UAS) to improve plant assessments of soybean iron deficiency chlorosis (IDC). The objective of this research was to use images collected from a UAS to measure IDC severity and determine the accuracy and precision of these predictions.

## Methods

### Plant material, location, and field design

**Fig. 1:**
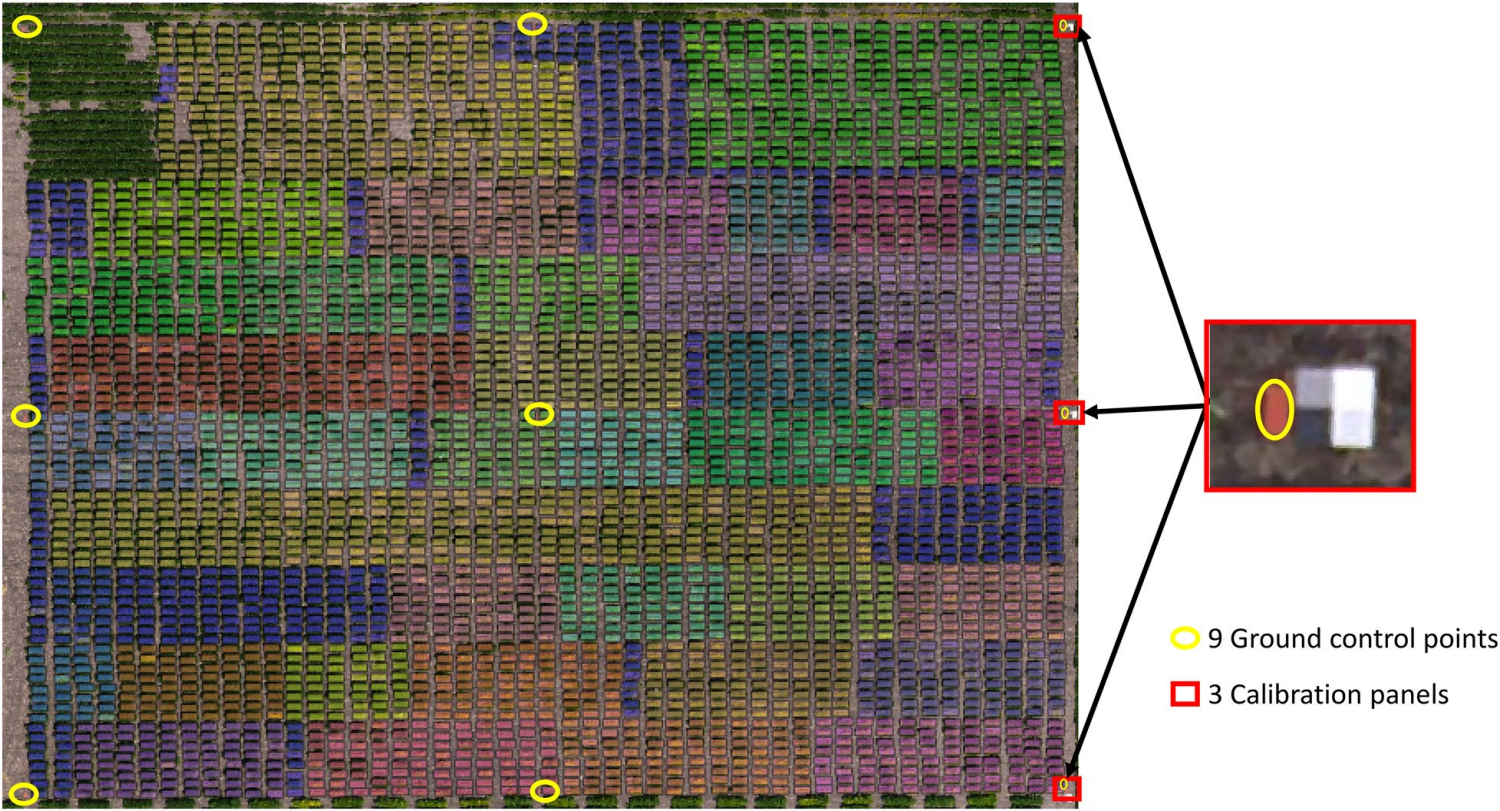
Soybean iron deficiency chlorosis testing field site location near Danvers, MN. The field used in this study is located in Six Grove Township, MN (45.274285, − 95.718046) in Swift County. The yellow circles highlight the nine ground control points in the field for geometric calibration and the red squares highlight the radiometric calibration panels. These panels were painted with four levels of grey for empirical line method calibration. Overlaid to the field orthomosaic is a vector file delineating the plot boundaries of 3386 soybean plots. Each plot boundary is colored based on the trial each plot belongs to. A total of 36 trials was grown

A series of 36 trials consisting of breeding lines at different stages in the UMN breeding program, ranging from the advanced yield trial stage to the regional trial and commercial testing stage, was used in this study (Fig. 1). Breeding lines belonged to maturity groups ranging from 00 to II. Individual trials consisted of breeding lines of similar relative maturity. The number of entries in each trial ranged from 16 to 80. Each plot (experimental unit) was planted as a single row 91 cm in length and 76.2 cm apart. Plots were arranged in a randomized complete block design with two replications. All plots (a total of 3824) were planted on June 1, 2017.

The location for this study was a field site near Danvers, MN (45.274285, − 95.718046) in Swift County. This field has a history of soybean IDC and has been rotated between corn and soybean for several years. Before planting, soil cores were taken and the soil was confirmed to have a pH in the range of 7.5 to 8.2, a range known to induce iron deficiency in soybeans [2, 8].

A total of nine ground control points (40 cm × 20 cm cement pavement blocks painted red) were placed in the field (Fig. 1). These were placed randomly throughout the field site location in such a way to cover the entire area of interest and remained in the field for the duration of the season. The ground control point coordinates were collected at one time-point during the summer using a Trimble Handheld GPS unit.

Reflectance calibration panels were also created for use in this project. A total of three calibration targets (2 feet by 2 feet matte boards) were made with each target consisting of four levels of grey—5%, 20%, 40%, and 55% reflectance painted with ‘black’, ‘iron mountain’ ‘flannel gray,’ and ‘silver bullet,’ and for each % reflectance respectively (BEHR paint brand, Santa Ana, California) Paint was mixed with a 50/50 mix by weight with barium sulfate to ensure a near Lambertian surface. A total of three layers of paint were used on top of one coat of primer. An ASD (Analytical Spectral Devices) Handheld 2: hand held VNIR spectroradiometer was used to measure the reflectance of the panels with the built-in halogen bulb for source lighting.

### IDC ground-based phenotyping

Soybean IDC was rated on July 12 and August 1, 2017, herein referred to as date 1 and date 2 respectively. On date 1, soybeans ranged in vegetative growth stages from V3–V6 and on date 2, soybeans ranged in growth stages from V6–R2. Soybeans were rated on both dates with a 1–5 visual rating scale. With this scale, a rating of “1” indicates a plot that is 100% green (no yellowing), a score of “2” indicates slight yellowing with some plants in the plot turning yellow, a score of “3” indicates moderate yellowing with most plants turning yellow in the plot, a score of “4” indicates intense yellowing where all plants are yellow and some are becoming stunted and necrotic, and a score of “5” indicates most severe IDC symptoms where the entire plot is damaged and dying or completely dead [6, 25]. Each plot was measured by an expert rater who understands IDC stress symptoms. This ground-based phenotyping served as the reference data in this study for training and validating models. In addition, 252 plots were scored independently by two trained raters for the assessment of inter-rater variability. These plots were chosen because they displayed variation in IDC severity because of their placement in a part of the field with optimal IDC stress for detecting differences between varieties.

### UAS platform, sensor, flight plan

In this study, a pipeline was created from image capture to image analysis. Table 1 highlights the major steps in this pipeline including (1) UAS image collection, (2) orthomosaic generation, and (3) image processing. In step 1, aerial data were collected on the same dates as ground based data (July 12 and August 1) with a DJI Inspire 1 equipped with a modified dual camera system, “Sentera Double 4 K agricultural” (Sentera Inc, Minneapolis, MN). The images were captured in 12.3 mega-pixel (MP) red (650 nm × 70 nm width), green (548 nm × 45 nm width), blue (446 nm × 60 nm width) and 12.3 MP red (650 nm × 70 nm width), green (548 nm × 45 nm width), Near Infrared (NIR, 840 nm × 20 nm width). Each camera has a 60° field of view. The UAS was flown using the autonomous flight mission planning of AgVault software. All missions were conducted at an altitude of 60.96 m with a UAS speed of 5 m/s and images captured with 70% end lap and side lap. The resulting images had a ground sampling distance of 1.6 cm. All flights were conducted within 2 h of solar noon to limit shadow effects.

**Table 1:** Pipeline for image capture and analysis for iron deficiency chlorosis assessment

Category	Step	Details
UAS image collection	Set up UAS	DJI Inspire 1 with Sentera Double 4 K sensor
	Prepare flight path	AgVault mobile app or Pix4D capture app
	Fly UAS for data collection	70% image overlap, 61 m altitude
Image orthomosaic using Pix4D	Initial processing	Key points extraction and matching, camera model optimization, geolocation
	Point cloud and mesh	Point densification and 3D textured mesh creation, insert ground control points
	Digital surface model, orthomosaic, and index	Creation of digital surface model, Orthomosaic, reflectance map, and index map
Image processing	Plant and soil classification	*k*-means clustering and recode to plant and soil
	Green, yellow, brown pixel classification	*k*-means clustering on masked canopy and recode to green, yellow, brown
	Neural network/random forest with ground data	Subset into training and validation sets, ground based data is response variable and green, yellow, brown pixel counts are used as features

The flight path is set up in Pix4D capture with 70% overlap of images. Individual photos are orthomosaiced in Pix4D and *k*-means clustering is used to mask the plants from the soil background. An additional classification of green, yellow, and brown pixels is performed on the plant objects. In QGIS, plots are defined, and the proportions of green, yellow, and brown pixels are extracted from each plot. Finally, predictions are made to correlate these three features with ground based visual score estimates rated on a one through 5 scoring system

### Image data processing

After image collection, image orthomosaics were generated using Pix4D Desktop (Pix4D, SA). The WGS 84 datum was used with a projected coordinate system of UTM zone 15 N. Images captured by the Sentera dual camera system were uploaded into the same project and given group names of “RGB” and “NIR” for the two sets of images respectively. For this study, only RGB data were used in image processing. The default processing options template, “Ag RGB,” was used for generating geo-referenced orthomosaics. This option generates mosaics from overlapping nadir images and outputs a full resolution GeoTIFF file and merges tiles. In addition, the “Ag RGB” processing template has faster processing speed and is compatible with RGB cameras.

Pix4D Processing occurs in three major steps including initial processing; point cloud and mesh; and digital surface model, orthomosaic, and index generation. Nine ground control points were input into the Pix4D project directly following the initial processing step using the ray cloud editor.

In step 3, orthomosaics were processed and data extracted from each plot. Orthomosaics were loaded into Erdas Imagine and unsupervised classification using *k*-means clustering into five classes was done on an indexed map of the ratio of red and green (R − G)/(R + G). After unsupervised classification, the five classes were manually grouped into the “plant” class and the “soil” class based on human observation of the five classes. The classes were recoded, and a mask was set based on the new classification.

Accuracy assessment of the plant and soil classification was conducted using Erdas Imagine software (Hexagon Geospatial, United States). The accuracy assessment toolkit was used to assign 1000 points across the field of interest. An equalized random sampling scheme was used to set 500 sampling points of soil and 500 of plant classified pixels. The reference data was created by visually assigning each point as plant or soil based on human interpretation of the non-classified original orthomosaic. Class values were hidden during the reference data collection in order to ensure unbiased values. An error matrix was used to compute the overall accuracy, producer accuracy, and user accuracy. The overall classification accuracy was computed by summing the major diagonal numbers (correctly classified) divided by the sum of all sample units in the entire matrix. The producer accuracy was computed by dividing the total number of correct sample units in each specific category by the total number of the specific sample units of reference data. The user accuracy was computed by dividing the total number of correct sample units in the specific category by the total number of sample units classified as the specific category.

The masked plant canopy was further classified to green, yellow, and brown plant pixels using an additional *k*-means clustering step. This was done to mimic how researchers typically rate IDC plots and was based previous studies [17, 19]. These features were then extracted using QGIS software (QGIS Geographic Information System. Open Source Geospatial Foundation Project. http://qgis.osgeo.org). In QGIS, a polygon shapefile was created where field plot polygons were used to identify each of the 3824 plots. The zonal statistics plugin was used to extract the proportion of green, yellow, and brown pixels in each plot.

Two modeling algorithms, neural network and random forest, were used to relate these three features to the ground based visual scores [26]. Both algorithms are available in the predictive analytics software package within JMP Pro (JMP^®^, Pro 10, SAS Institute Inc., Cary, NC, 1989–2007). In both cases, the IDC visual score data was treated as a character and 77% of the data, at random, was used to train the models and 33% of the data, at random, was used for validation. The random forest used 100 trees in the forest with 10 minimum splits per tree. The neural network was run using default settings in JMP software. The hidden layer structure used 3 TanH functions and a learning rate of 0.1. Results are reported in a confusion matrix.

Each of the 36 trials in the field were analyzed using a linear model including the IDC score as the response variable, a fixed effect for entry, a fixed effect for block, and a random residual. The least significant difference (LSD) was calculated by multiplying the square root of the mean square error from the statistical model by the 0.2 t-value for each test. The LSD was calculated for all 36 tests on ground-based UAS scores for both dates of data collection.

## Results and discussion

### Ground-based phenotyping

**Fig. 2:**
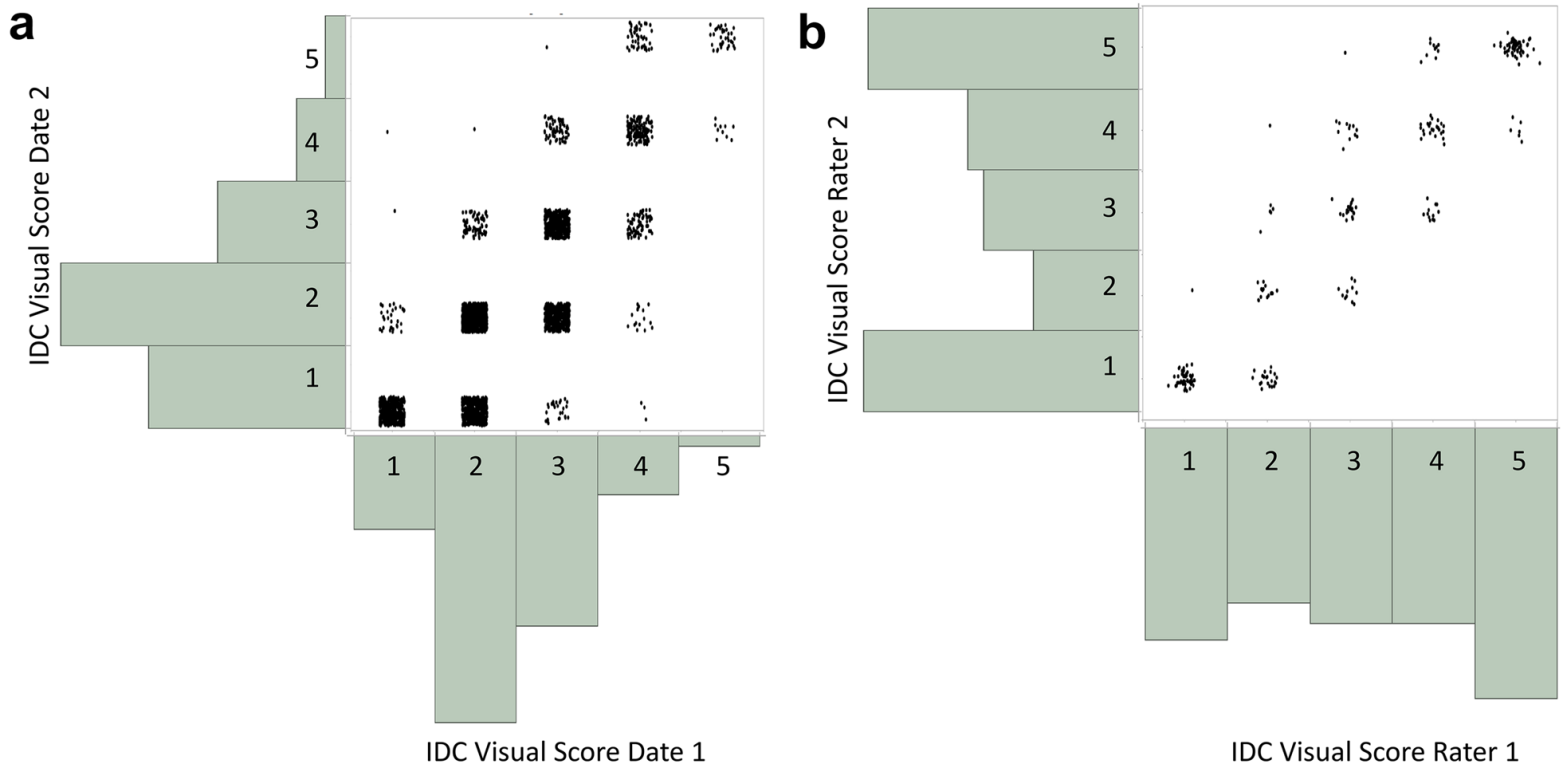
Relationships between two dates of iron deficiency chlorosis (IDC) severity (**a**) and two separate raters scoring plots (**b**). IDC severity was measured on a total of 3386 plots on both July 12 and August 01. The correlation of ratings between date 1 and date 2 was found to be 0.80. A subset of 252 plots were measured by two independent raters on date 1. The correlation of ratings between raters was found to be 0.93

A total of 3386 plots were assessed for severity to IDC stress on a 1–5 scale on two separate dates (July 12 and August 1, 2017). The distributions of each of the two dates of data, as well as their relationships, can be seen in Fig. 2. In total, 15%, 45%, 30%, 9%, and 1% were rated 1–5, respectively, on date 1. On date 2 30%, 41%, 18%, 8%, and 3% were rated 1–5, respectively. In total, there was a much higher abundance of entries given a score of “1” on date 2 as compared to date 1. Nevertheless, the overall average score in the field was 2.1 on both dates of visual scoring. The comparison between raters was also investigated on a subset of 252 plots. For these plots, two people independently scored the plots to provide an estimate of inter rater variability in visual scores. The correlation between rater 1 and rater 2 was 0.93.

### UAS imagery for IDC phenotyping

**Fig. 3:**
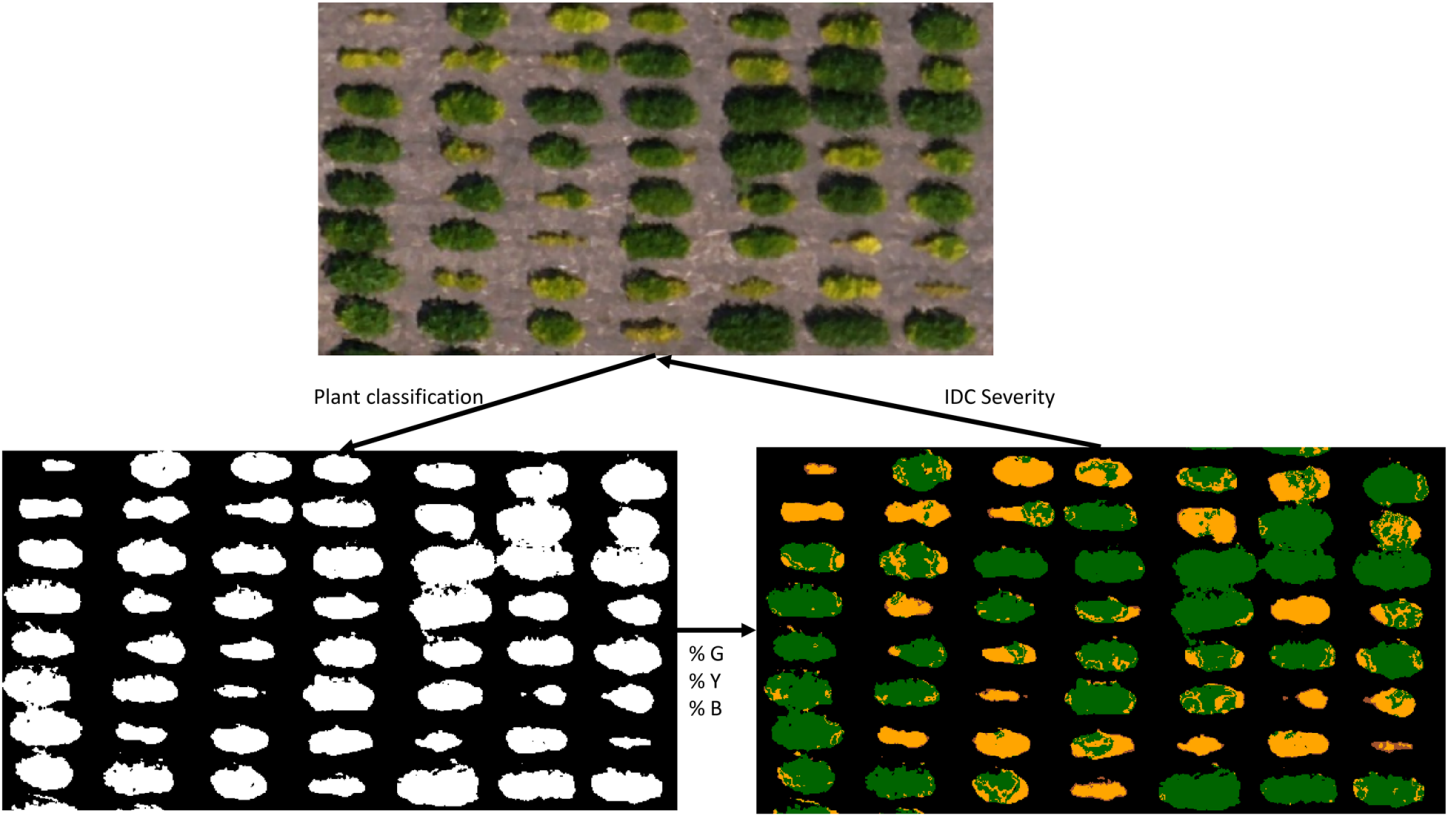
Iron deficiency chlorosis classification. The Orthomosaic (top) is first classified into plant and soil pixels (bottom left). The plant pixels are then classified in a second step to green pixels (%G), yellow pixels (%Y), and brown pixels (%B) (bottom right). These features are then related back to ground-based visual scores through random forest and neural network models to classify tolerant and susceptible plots

In the first step of the image analysis pipeline, plant canopy was masked from the soil using *k*-means clustering (Fig. 3), an unsupervised machine learning approach that has been successfully used in many agriculture applications [27, 28]. This approach resulted in an overall classification accuracy of greater than 95%. The user accuracy was 94% and 97% for soil and plant classification, respectively. Table 2 is a confusion matrix highlighting the results of the accuracy assessment of this classification.

**Table 2:** Accuracy assessment of pixel-based classification method for plant and soil classification

	Reference data			User accuracy (%)
	Soil	Plant	Row total	
Predicted data				
Soil	*469*	31	500	93.8
Plant	13	*487*	500	97.4
Column total	482	518	1000	
Producer accuracy (%)	97.3	94.0		
Overall accuracy (%) = 95.6				

One thousand random points were generated and placed on the orthomosaiced image using the equalized random sampling method. The predicted data was generated from *k*-means clustering and the reference data was manually created using visual observations of the images. Accuracy assessment results were generated using ERDAS IMAGINE software. An overall classification of 95.6% was achieved

The diagonal elements are italicized to highlight the number of correctly classified pixels in terms of plant or soil classifications

Previous studies have utilized a variety of techniques to mask plant canopies. In a publication by Yu et al., for example, plant and soil classification was achieved using a random forest model and resulted in an accuracy of 99.9% [23]. The results here had an accuracy of 95.6%, however, plants under chlorosis or necrosis stress result in spectral properties very similar to that of the soil background. In addition, many misclassification errors occurred at the edges of the canopy where soil and plant canopy pixels were overlapping. One advantage of this study, however, is the ability to classify plant and soil without training data. The unsupervised classification approach using *k*-means clustering resulted in pixels with similar spectral values being clustered together. This clustering occurs with no former knowledge or input, and can often provide new insights into the data [18]. With supervised approaches such as the random forest, a training data set must be generated by manually interpreting the classes pixels belong to. Other studies have used thresholding approaches to differentiate plants from soil. In these scenarios, the image is often thresholded based on the hue, saturation, value format of the image or based on indices or ratios of different color bands [17, 24, 29].

The percent green, yellow, and brown pixels were also classified using a similar approach as the plant canopy classification (Fig. 3). These features were used in two different machine learning models (random forest and neural network) to predict IDC severity scores. The confusion matrices for the random forest models for date 1 and date 2 are shown in Tables 3 and 4 for date 1 and date 2, respectively. Overall, the classification accuracy was 68% for date 1 and 77% for date 2. The increase in classification accuracy between date 1 and date 2 is likely the result of the size of the plants, with more pixels representing plant material at date 2 than at date 1.

**Table 3:** Random forest confusion matrix for date 1 of data collection (July 12)

	Reference data				
	1 (%)	2 (%)	3 (%)	4 (%)	5 (%)
Predicted data					
1	*76.4*	22.8	0	0.8	0
2	14.3	*68.2*	16.1	1.2	0.2
3	0.8	19.2	*65.3*	14.7	0
4	0	3.9	19.7	*61.9*	14.5
5	0	0	0	100	*0*
Overall accuracy (%) = 68					

The % green, % yellow, and % brown pixels from each of the research plots were used as features in a random forest model. This confusion matrix shows how well the random forest model predicted the iron deficiency chlorosis (IDC) score from ground-based reference data where each plot was rated on a one through five scale. The overall accuracy was 68%

The diagonal elements are italicized to highlight the percentage of correctly classified field plots in terms of IDC score

**Table 4:** Random forest confusion matrix for date 2 of data collection (August 1)

	Reference data				
	1 (%)	2 (%)	3 (%)	4 (%)	5 (%)
Predicted data					
1	*85.1*	14.9	0	0	0
2	10.4	*79.6*	9.3	0.5	0.2
3	0.2	13	*74*	12	0.8
4	0	0.6	9.9	*73.6*	15.9
5	0	0	3.1	18.4	*78.5*
Overall accuracy (%) = 77					

The % green, % yellow, and % brown pixels from each of the research plots were used as features in a random forest model. This confusion matrix shows how well the random forest model predicted the iron deficiency chlorosis (IDC) score from ground*-*based reference data where each plot was rated on a one through five scale. The overall accuracy was 77%

The diagonal elements are italicized to highlight the percentage of correctly classified field plots in terms of IDC score

The average canopy coverage of each plot can indicate the overall biomass and light interception of the plots. Canopy coverage was thus calculated as the ratio of “plant classified pixels” to “soil plus plant classified pixels” and compared across the five different severity classes. The average canopy coverage for each IDC class was as follows: class 1 = 0.264, class 2 = 0.246, class 3 = 0.189, class 4 = 0.096, and class 5 = 0.039. Each of these levels was considered significantly different from each other class at the 0.05 probability level using Tukey’s honest significant difference, indicating the IDC score explained variation in plot biomass.

#### UAS imagery is a reliable measure for detecting differences among entries

**Fig. 4:**
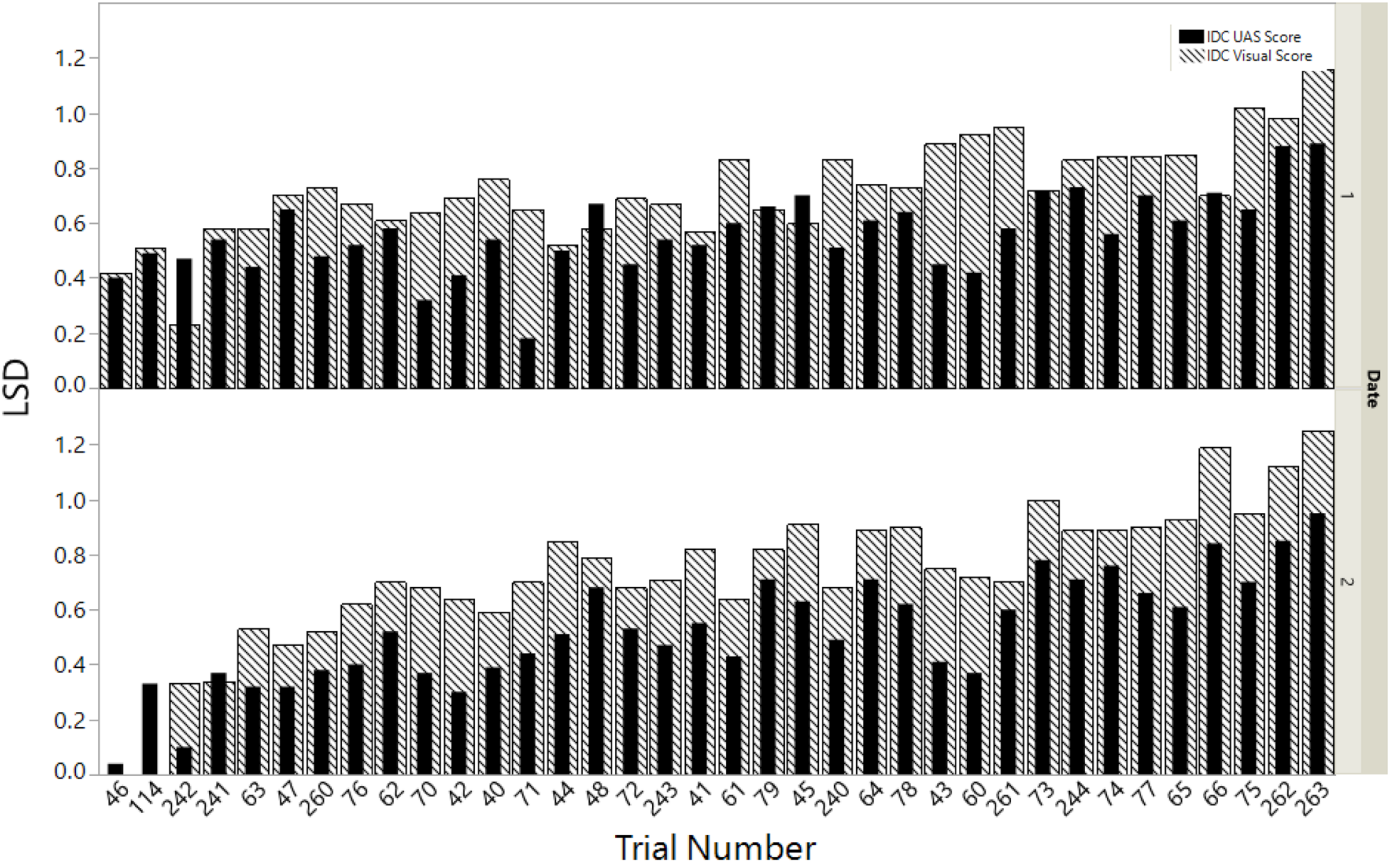
A total of 36 trials consisting of soybean breeding lines, each arranged in a randomized complete block design in the field, were analyzed using a linear model for both visual score and unmanned aircraft system (UAS) predicted values on both dates (July 12 and August 01). Bars indicate the least significant difference (LSD) values to separate mean scores of breeding lines for each trial in the field. In 31 of the 36 trials on date 1 (top) and 33 of 36 trials on date 2 (bottom), the LSD was decreased when using the UAS predicted IDC scores (black inside bars in the linear model compared to the visual scores (dashed outside bars)

Another method to test the suitability of the UAS scores is to test if the scores can differentiate breeding lines in the field. A common procedure for this assessment is to calculate the LSD for comparing performances of breeding lines in typical randomized field trials. For reporting of variety trials, a relaxed significance threshold (e.g., P < 0.20) is commonly used for declaring differences among varieties to increase power and reduce type II errors. Based on this, if the LSD is low, the precision of the data is higher, and the researcher is better able determine differences among breeding lines. The LSD values from the ground-based data and the UAS data were compared among the 36 experimental trials, or sets of lines, for both dates of data collection. In 32 of the 36 trials on date 1 and 33 of 36 trials on date 2, the LSD was smaller when using the UAS IDC scores in the linear model compared to the visual scores (Fig. 4). There was an average 0.15- and 0.20-point reduction in LSD (ranging from 0.35 increase to a 0.50 decrease) with UAS scores compared to the visual scores for date 1 and date 2 respectively. This reduction in LSD indicates that the UAS data is more precise than visual scores and offers appropriate information for use in a breeding program.

Many previous studies have shown great success and accuracy in collecting phenotypic traits from unmanned aircraft systems both in soybeans and in other crops [22, 23, 30, 31]. In addition, previous studies have successfully used image-based methods for classifying and quantifying soybean IDC stress [17, 32]. However, to date, no study has demonstrated combining the powers of high throughput image collection from unmanned aircraft systems with image-based classification for IDC.

#### UAS imagery compared to other methods for IDC assessment and limitations

Previous studies have tested the use of image analysis procedures to quantify IDC stress [17–19]. Their approaches achieved a mean per class accuracy of ~ 96% and 81%. While the previous studies showed relatively high accuracy, the throughput of taking photos from a tripod or push cart is much lower in comparison to a UAS. Spatial resolution achieved, however, was much higher (6 pixels/mm from tripod compared to 1.6 cm/pixel from the UAS). This decrease in spatial resolution resulted in blending of pixels and thus less resolution to depict subtle changes of individual leaves becoming chlorotic.

For this study, a flight path was chosen to cover a large area within one UAS battery life, and to limit variation in sun positioning and cloud shadows during flight, as these variables are known to present problems with image analysis applications. Future research should be done to test if an increase in spatial resolution through improved camera sensors or lower flight altitudes would improve predictive abilities. Care should be taken to ensure that flights conducted limit shadowing caused by soybean plots by flying near solar noon, and that the cloud cover during flights be consistent—full cloud cover or no cloud cover is optimum. The system presented in this paper reduced flight time by flying at a higher altitude and will allow researchers to achieve a much higher temporal assessment of IDC severity as well as the ability to rate more plots in any given growing season.

Additionally, the confusion matrices show that many of the misclassified scores are mostly between nearby classes. If a breeder simply wishes to select against scores of 4 or 5, for example, the UAS image-based scores would be very adequate for breeding purposes. To test this, scores of 4 and 5 were binned together as “high stress” and scores of 1, 2, and 3 were binned together as “low stress.” The overall accuracy of the random forest model in correctly placing the entries into these two categories was 89%.

A final caution that researchers should consider in employing UAS imagery for IDC detection is that other soybean stresses or field variables may mimic IDC symptoms detected by image analyses. One major biotic factor to soybean production, for example, is soybean cyst nematode. Research plots may experience stunting and chlorosis from nematode presence, which could be rated as IDC susceptible using this system. In addition, healthy weeds in the field would be detected by this system as healthy vegetation, and thus, healthy plots. These cautions were addressed by site selection with limited known off-target stressors and by proper weed control prior to all UAS flights, and should be considered by researchers wishing to employ this technology.

## Conclusions

In this study, we achieved high efficiency in collecting data with autonomous UAS flights, greater than 77% accuracy in classifying plots on a 1–5 severity scale, and confidence in this system for IDC assessment through an average reduction in LSD values across a series of experimental trials. This method is high-throughput, objective, and more precise than traditional ground based visual assessments. In addition, it allows researchers to collect information from more plots in a given year and at a much higher temporal frequency than before.

## Data Availability

The UAS Images and remotely sensed data used in this study are available upon the approval of Aaron Lorenz from the University of Minnesota Department of Agronomy and Plant Genetics. The ground-based IDC severity data in this study is available upon the approval of Aaron Lorenz from the University of Minnesota Department of Agronomy and Plant Genetics.

## Abbreviations

IDC: iron deficiency chlorosis
UAS: unmanned aircraft system
NIR: near infrared
HTP: high throughput phenotyping
LSD: least significant difference
