# Correction to: Soybean iron deficiency chlorosis high throughput phenotyping using an unmanned aircraft system

**DOI:** 10.1186/s13007-019-0495-8

**Published:** 2019-10-10

**Authors:** Austin A. Dobbels, Aaron J. Lorenz

**Affiliations:** Department of Agronomy and Plant Genetics, University of Minnesota, 1991 Upper Buford Circle, 411 Borlaug Hall, St. Paul, MN 55108 USA

## Correction to: Plant Methods (2019) 15:97 10.1186/s13007-019-0478-9

In the original article [1], under the subheading “Image data processing”, last paragraph, last sentence that reads as “The least …… data collection” was incorrectly published. The correct sentence should read as “Least-significant differences (P < 0.20) were calculated for all 36 trials on both ground-based and UAS-image based scores for both dates of data collection.” The original article has been corrected.
